# Robust Control of Distribution Static Compensator in Self-Excited Induction Generator-Based Wind Energy Systems Under Sensor Failures and Abnormal Load Conditions

**DOI:** 10.3390/s26092902

**Published:** 2026-05-06

**Authors:** Ali Sait Özer, Hulusi Karaca

**Affiliations:** 1 Department of Control and Automation Technology, Konya Technical University, Konya 42250, Türkiye; asozer@ktun.edu.tr; 2 Department of Electrical and Electronics Engineering, Selçuk University, Konya 42130, Türkiye

**Keywords:** ADFOGI-PLL, DSTATCOM, SEIG, DC offset, sensor-induced errors, harmonic estimation, power quality

## Abstract

**Highlights:**

**What are the main findings?**

- An ADFOGI-based control algorithm is developed to enhance voltage and frequency regulation of SEIG–DSTATCOM systems under distorted conditions.
- The proposed method ensures robustness against sensor-induced DC offset errors.

**What are the implications of the main findings?**

- The proposed approach improves measurement robustness against sensor-induced errors, enabling more reliable reference current generation in practical applications.
- The method offers a promising solution for improving power quality in renewable energy-based standalone systems while complying with IEEE-519-2022 standards.

**Abstract:**

Self-excited induction generators (SEIGs) used in wind energy systems suffer from poor voltage and frequency regulation due to varying active/reactive power demands of nonlinear and unbalanced loads. The distribution static compensator (DSTATCOM) provides an effective solution through reactive power support and harmonic mitigation. However, its performance strongly depends on the robustness of the control algorithm against harmonics, load imbalance, and sensor-induced measurement errors such as DC offset, which degrade reference current generation. This study proposes an Advanced Dual Fourth-Order Generalized Integrator (ADFOGI)-based control algorithm to improve voltage and frequency regulation of SEIG–DSTATCOM systems under such adverse conditions. The proposed method inherently rejects DC offset components and enables accurate reference current generation even under severe harmonic distortion, load imbalance, and transient disturbances. The effectiveness of the approach is validated on an OPAL-RT real-time platform under three scenarios: nonlinear load, unbalanced nonlinear load, and one-phase open-circuit condition, where DC offset is intentionally introduced to emulate sensor errors. Under the most severe case, where load current THD reaches 16.23%, SEIG current THD is reduced to 3.71% and voltage THD to 1.66%. In all scenarios, harmonic levels remain below the IEEE-519-2022 limit of 5%, confirming the robustness and effectiveness of the proposed control strategy.

## 1. Introduction

The increase in global energy demand and the depletion of fossil fuel reserves have accelerated the shift towards renewable energy sources [1]. However, the integration of renewable sources into modern power systems raises new stability and control issues [2]. Wind energy, on the other hand, is widely preferred, especially in isolated systems, due to its low operating costs and technological maturity [3,4].

Maintaining stable power quality in wind energy systems depends not only on the turbine performance but also on the characteristics of the generator type used. Synchronous generators with good voltage regulation capabilities are commonly used in such applications. However, owing to their brush and ring structures, they require high maintenance and carry a risk of failure. High-efficiency permanent magnet synchronous generators (PMSG) are also preferred. However, their high cost, sensitivity to temperature, and risk of demagnetization during long-term operation are significant disadvantages of PMSG [5]. On the other hand, wound-rotor asynchronous generators are mechanically more complex and have a high risk of failure, despite their flexibility in reactive power control [6]. For this reason, simple, robust, and low-maintenance generator structures are preferred in small and medium-scale wind turbine applications [7,8]. Self-excited asynchronous generators (SEIG) stand out as a suitable option due to advantages such as their brushless design, low cost, and ability to protect themselves against short-circuit conditions [5,9].

The widespread use of nonlinear loads in modern isolated wind energy systems leads to increased current harmonics, voltage waveform distortions, interphase imbalances, and variable active/reactive power demands [10]. In self-excited induction generators (SEIGs), the terminal voltage and frequency are inherently determined by the balance between the machine magnetization characteristics, excitation capacitance, and load conditions.

Unlike synchronous generators, SEIGs lack an independent excitation control mechanism; therefore, their voltage regulation is highly dependent on the reactive power supplied by the excitation capacitor and the connected load. Any variation in load impedance, particularly under nonlinear or unbalanced conditions, alters the reactive power balance, leading to fluctuations in the air-gap flux and consequently causing voltage instability.

Similarly, the frequency of SEIG is directly influenced by the balance between mechanical input power and electrical output power. Sudden changes in load demand disturb this balance, resulting in frequency deviations. Furthermore, nonlinear loads inject harmonic currents into the system, which distorts the stator flux and degrades the electromagnetic coupling within the machine, further deteriorating both voltage and frequency stability.

In addition to load-related effects, measurement imperfections such as sensor-induced DC offset introduce low-frequency components into the measured signals. These components can propagate through the control loop, leading to inaccurate reference current generation and incorrect synchronization, which further exacerbates instability and power quality degradation [11].

Therefore, due to the combined effects of reactive power dependency, harmonic distortion, load imbalance, and measurement-induced errors, SEIG-based systems exhibit inherently weak voltage and frequency regulation capability, particularly under practical operating conditions.

Various compensation methods have been used to reduce the power quality problems that arise in SEIG-based isolated systems. Although passive filters offer a low-cost option for harmonic attenuation, they are incompatible with load changes and carry the risk of resonance [12]. One of the most common conventional solutions is the static var compensator (SVC). Although SVCs are effective in voltage support and reactive power balancing, they cannot adapt quickly to load changes, are insufficient in suppressing harmonics, and exhibit limited performance in phase imbalances [13]. Although dynamic voltage regulators (DVRs) are effective in events such as voltage dips or surges, they cannot provide a comprehensive solution in SEIG systems due to their operation based on the series connection principle and their inability to compensate for current harmonics [14].

DSTATCOM provides reactive power support while actively suppressing harmonic currents, eliminating phase imbalances, and offering fast dynamic response capability [15]. Therefore, the DSTATCOM structure stands out as one of the most suitable solutions for SEIG-based isolated systems [16]. DSTATCOM requires an advanced control algorithm, particularly to overcome SEIG’s weak voltage regulation problem and to provide stable voltage-frequency regulation under variable load conditions [17].

Different synchronization and reference current generation methods were developed for DSTATCOM control to meet specific performance requirements, such as steady-state synchronization, harmonic mitigation, separation of positive and negative sequence components under unbalanced conditions, and fast dynamic response.

Conventional phase-locked loop techniques, including the synchronous reference frame phase-locked loop (SRF-PLL) [18,19] are among the most used synchronization approaches in literature. Despite being preferred for many years due to its simple structure and success in steady-state synchronization, its sensitivity to grid imbalances and harmonics are significant disadvantages.

Generalized integrator-based techniques, including second-order generalized integrator frequency-locked loop (SOGI-FLL) [20], dual second-order generalized integrator phase-locked loop (DSOGI-PLL) [21], and multiple second-order generalized integrator (MSOGI) structures [22], were developed. SOGI-FLL-based methods are successful in separating positive and negative components but are overly sensitive to coefficient adjustments and have a high computational load. DSOGI-PLL operates stably in unbalanced systems, but it cannot sufficiently filter dominant harmonics due to its low-order structure. MSOGI structures were proposed to improve filtering performance. Since it contains multiple filter blocks, the computational load increases significantly, making it impractical for embedded system applications.

Delay-based synchronization techniques, including delayed signal cancellation (DSC)-PLL [23] and moving average filter (MAF)-based PLL [24] methods, were proposed to improve harmonic filtering performance. In delayed signal cancelation (DSC)-based PLL approaches, the use of multiple delay operators causes the PLL loop to slow down in transient mode. In moving average filter (MAF)-based PLL methods, as the window length increases, harmonics are filtered better, but this time the dynamic response slows down significantly. Although quasi-type-1 (QT1)-PLL provides a solution to this problem, its sensitivity to deviations in the grid frequency limits its performance [23].

Adaptive and intelligent techniques, including least mean square (LMS) [25], least mean fourth (LMF) [26], momentum least mean square (MLMS) [27], quasi-Newton least mean fourth (QNLMF) [28], adaptive least-error squares phase-locked loop (ALES-PLL) [29], genetic algorithm-based fuzzy logic control (GA-FLC) [30], fuzzy logic-based STATCOM control strategies under abnormal conditions [31] neural network-based LMS/LMF hybrid methods [32], amplitude adaptive notch filter (AANF) techniques [33], and deep learning or ANN-based methods [34], were proposed to address adaptive harmonic mitigation and nonlinear behavior compensation. However, most of these methods have limitations such as high computational cost, parameter tuning sensitivity, real-time applicability constraints, or instability under unbalanced/harmonic conditions. In particular, deep learning or ANN-based methods, while capable of learning complex nonlinear behaviors, are not suitable for practical real-time applications due to their high computational requirements and need for large datasets.

Although all these methods offer certain advantages in power quality applications, they may not provide stable and accurate performance under challenging operating conditions such as high harmonic content, sudden phase changes, unbalanced load conditions, the presence of DC offset, and frequency fluctuations. Therefore, a more advanced control algorithm is needed that possesses strong harmonic suppression capabilities, can accurately separate the positive sequence component under unbalanced conditions, and can respond quickly in transient regimes.

In this study, an Advanced Dual Fourth-Order Generalized Integrator (ADFOGI)–based DSTATCOM control method is proposed. The proposed method targets the reduction of harmonic distortion in SEIG currents and voltages in SEIG-based wind energy systems operating under nonlinear loads, unbalanced and nonlinear loads, and one-phase open-circuit conditions in the presence of DC offset. In addition, the method aims to maintain the system frequency at 50 Hz and the phase voltage at 326.5 V (peak value) under these challenging operating conditions. The proposed control strategy is validated on the OPAL-RT real-time platform.

The proposed ADFOGI-based DSTATCOM control method preserves the inherent advantages of the conventional DTOGI structure while being redesigned to achieve immunity against DC offset components, which typically cause significant issues within the control loop. Additionally, the proposed method is effective under unbalanced operating conditions due to its enhanced negative-sequence suppression capability, and it provides good harmonic filtering performance. Thus, it becomes possible to extract the fundamental component more accurately and determine the reference currents more stably under nonlinear and unbalanced load conditions in the presence of DC offset.

The effectiveness of the proposed ADFOGI-based control method is evaluated under three challenging operating conditions, including the DC offset effect caused by sensor and measurement errors. In the first scenario, a nonlinear load is connected to the system, and a DC offset is added to the measurements. In the second scenario, the load is both unbalanced and nonlinear, also containing a DC offset. Furthermore, during this test, the load is increased to examine the control structure’s response to dynamic changes. In the third scenario, one phase of the load is removed from the three-phase system, and DC offset is again added to the measurements. These three different conditions are selected to evaluate the algorithm’s resilience against harmonic distortion, unbalanced loads, and DC offset errors that may be encountered in real systems.

In all scenarios, the proposed ADFOGI-based control structure suppresses DC offset components, accurately extracts the fundamental component, and reduces harmonic and negative-sequence distortions caused by unbalanced loads. Also, it maintains the total harmonic distortions (THDs) of SEIG currents and SEIG voltages (PCC voltages) well below the 5% limit specified in IEEE 519-2022 [35] under all three operating conditions.

The system maintains voltage and frequency stability even under severe operating conditions such as high harmonic content, unbalanced load currents, and one-phase open-circuit.

The results clearly demonstrate that the ADFOGI-based control structure provides accurate and stable performance for DSTATCOM applications under practical operating conditions.

The main contributions of the proposed ADFOGI-based control method can be summarized as follows:

✓ It provides inherent immunity to sensor-induced DC offset errors, ensuring accurate synchronization and reference current generation.
✓ It significantly improves power quality by reducing SEIG current THD from 16.23% to 3.71% and voltage THD to 1.66%, satisfying IEEE 519-2022 limits.
✓ It maintains stable voltage and frequency under nonlinear, unbalanced, and one-phase open-circuit conditions with DC offset.
✓ It offers a practical and real-time applicable control structure validated on the OPAL-RT platform.

The following sections are organized as follows. Section 2 presents the configuration of the DSTATCOM-based SEIG system together with its main components and parameters. Section 3 introduces the proposed ADFOGI-PLL-based control strategy in detail, including its structure, parameter design, and stability analysis. Section 4 discusses the real-time experimental results obtained under various operating conditions, including nonlinear, unbalanced, and one-phase open-circuit scenarios with DC offset. Finally, Section 5 summarizes the main findings and discusses potential directions for future research.

## 2. DSTATCOM-Based SEIG System

**Figure 1:**
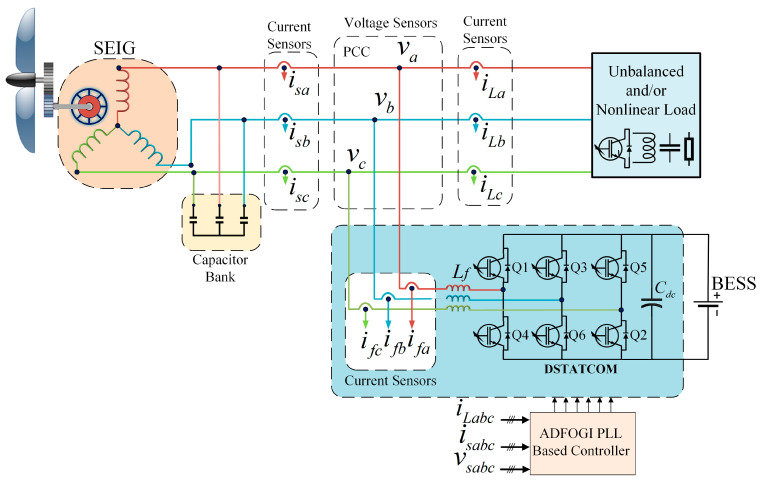
Schematic of DSTATCOM-based SEIG system.

Figure 1 shows the DSTATCOM-based SEIG power system supplying unbalanced and nonlinear loads. SEIG is commonly mechanically driven by a horizontal-axis wind turbine and requires an external reactive power source to generate voltage. During the initial excitation, since the DSTATCOM is not yet in operation, the necessary reactive power is supplied via excitation capacitors connected to the generator terminals.

DSTATCOM, connected in parallel to the point of common coupling (PCC), is activated to meet the additional reactive power demand arising from changes in operating conditions on both the generator and load sides. Its structure includes a voltage source converter (VSC), a three-phase coupling inductor (*L*), and a DC bus capacitor (*C_dc_*). The high-frequency IGBT-based converter ensures fast response, enhancing the overall stability and reliability of the system. The battery energy storage system (BESS) integrated into the system contributes to ensuring the voltage stability of the DC bus and improves the transient performance of the DSTATCOM by balancing the power flow during sudden load changes. In addition, the BESS supports the SEIG in producing voltage stably at a nominal frequency of 50 Hz.

The basic design parameters of the SEIG and DSTATCOM used in the study are given in Table 1. The calculation of these parameters is detailed in Appendix A. The proposed system structure was also tested and validated on an Opal-RT-based hardware-in-the-loop (HIL) platform.

## 3. Proposed ADFOGI-PLL-Based Control Method

**Figure 2:**
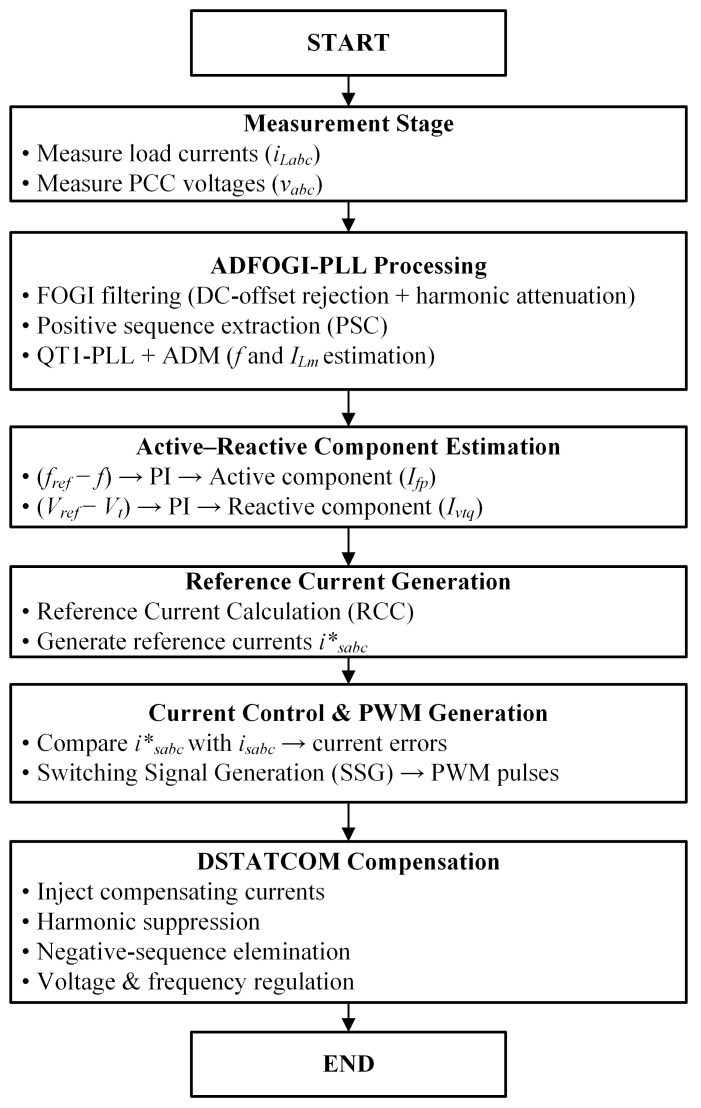
Algorithmic flowchart of the proposed ADFOGI-PLL-based DSTATCOM control strategy.

To provide an overview of the proposed control method, the algorithmic flow of the ADFOGI-PLL-based DSTATCOM control strategy is shown in Figure 2. The flowchart presents the sequential operation of the control algorithm, starting from signal measurement, including ADFOGI-PLL-based synchronization and fundamental component extraction, active and reactive component estimation via PI controllers, reference current generation using unit voltage templates, and PWM-based switching signal generation.

**Figure 3:**
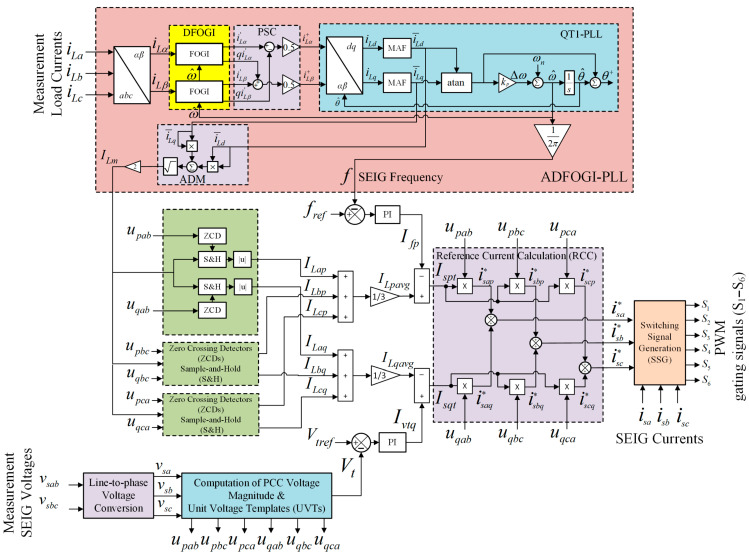
Proposed ADFOGI-PLL-based control technique.

Figure 3 shows the detailed structure of the proposed ADFOGI-PLL-based DSTATCOM control method. The control process consists of synchronization, power component extraction, reference current generation, and switching signal production stages. In the first stage, load currents and SEIG terminal voltages are measured. The measured load currents are processed by the ADFOGI-PLL structure. Using the fourth-order generalized integrator structure, the fundamental component of the load currents is extracted, and the SEIG frequency (f) and peak load current value ($I_{Lm}$) are estimated. The high order structure provides stronger harmonic filtering and eliminates DC offset components.

The estimated frequency is compared with the reference frequency, and the required active power component ($I_{fp}$) is determined through the PI controller to maintain frequency stability. Similarly, SEIG voltages are used to determine the reactive power component ($I_{vtq}$) required to regulate the terminal voltage magnitude.

In the next stage, the required active and reactive power components are converted into reference current components. These reference currents ($i_{sa}^{*}$, $i_{sb}^{*}$, and $i_{sc}^{*}$) are generated using the unit voltage templates obtained from SEIG voltages. The generated reference currents are compared with the measured SEIG currents.

Finally, the current error signals are processed by the switching signal generation block to produce PWM gating signals for the voltage source converter (VSC). Through this process, DSTATCOM injects the required compensation currents into the system to suppress harmonics, eliminate negative-sequence components, and maintain voltage and frequency at their reference values.

The proposed ADFOGI-PLL will be discussed in detail in the next section.

### 3.1. Structure of Proposed ADFOGI-PLL

The block structure of the proposed ADFOGI-PLL is shown in Figure 3. The proposed ADFOGI-PLL is an advanced structure of DTOGI-PLLs [18,36]. The ADFOGI-PLL employs two fourth-order generalized integrators (FOGIs)-based band-pass filter after abc/αβ transformation to filter out the load currents.

**Figure 4:**
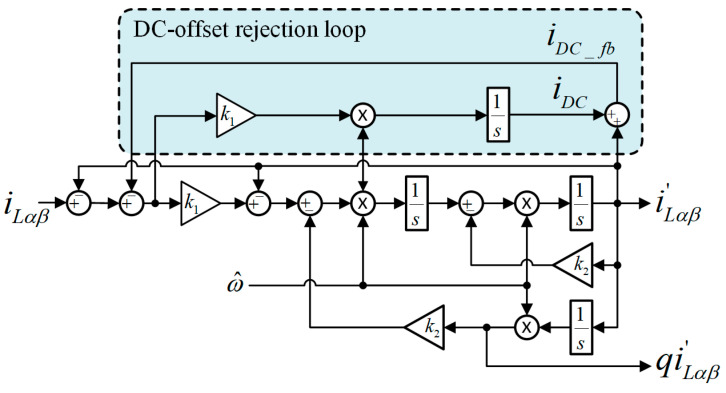
The block pattern of the FOGI filter with the DC offset rejection loop.

As observed from Figure 3, each FOGI is responsible for obtaining the filtered load currents (i.e., $i_{L\alpha }^{\prime }$ and $i_{L\beta }^{\prime }$) and their quadrature currents (i.e., $qi_{L\alpha }^{\prime }$ and $qi_{L\beta }^{\prime }$). The block pattern of the FOGI filter with DC offset rejection loop is illustrated in Figure 4. Considering this block diagram, the transfer functions of the FOGI in continuous-time domain can be derived, where R(s) is the in-phase transfer function of the input, and Q(s) is the quadrature transfer function of the input. In Equations (1) and (2), $\hat{\omega }$ is the detected angular frequency and the proportional gains $k_{1}$ and $k_{2}$ denote the parameters of the FOGI. The detected frequency $\hat{\omega }$ is fed back into each FOGI to adapt the frequency changes, as can be seen in Figure 3.

(1) $$R(s)=\frac{i_{L\alpha ,L\beta }^{\prime }}{i_{L\alpha ,L\beta }}=\frac{k_{1}{\hat{\omega }}^{2}s^{2}}{s^{4}+(k_{1}+k_{2})\hat{\omega }s^{3}+(1+2k_{1}+k_{1}k_{2}){\hat{\omega }}^{2}s^{2}+(k_{1}+k_{2}){\hat{\omega }}^{3}s+k_{1}k_{2}{\hat{\omega }}^{4}}$$

(2) $$Q(s)=\frac{qi_{L\alpha ,L\beta }^{\prime }}{i_{L\alpha ,L\beta }}=\frac{\hat{\omega }}{s}R(s)=\frac{k_{1}{\hat{\omega }}^{3}s}{s^{4}+(k_{1}+k_{2})\hat{\omega }s^{3}+(1+2k_{1}+k_{1}k_{2}){\hat{\omega }}^{2}s^{2}+(k_{1}+k_{2}){\hat{\omega }}^{3}s+k_{1}k_{2}{\hat{\omega }}^{4}}$$

On the other hand, the gains $k_{1}$ and $k_{2}$ affect the performance of the FOGI filter.

**Figure 5:**
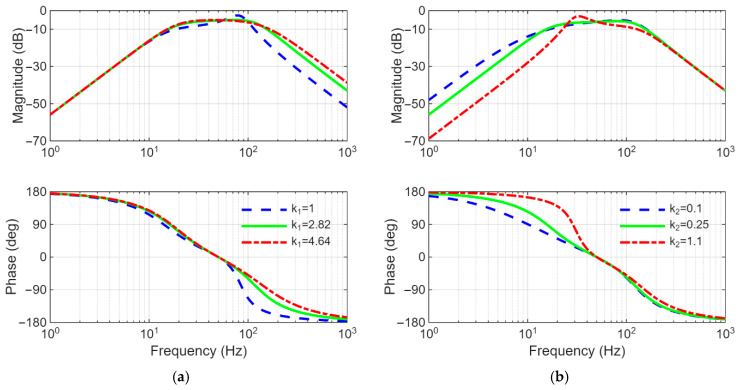
Bode plots of R(s) in Equation (1) (**a**) for different values of $k_{1}$ (here, $k_{2}=0.25$), (**b**) for different values of $k_{2}$ (here, $k_{1}=2.82$).

**Figure 6:**
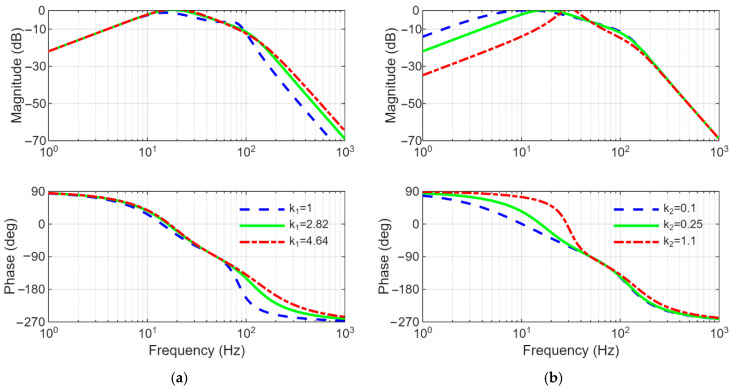
Bode plots of Q(s) in Equation (2) (**a**) for different values of $k_{1}$ (here, $k_{2}=0.25$), (**b**) for different values of $k_{2}$ (here, $k_{1}=2.82$).

$k_{1}$ determines the bandwidth of the filter while $k_{2}$ changes the dynamic response of the filter. To visualize this case, the Bode plots of Equations (1) and (2) are illustrated in Figure 5 and Figure 6, respectively. As shown in Figure 5a and Figure 6a, increasing in $k_{1}$ expands the bandwidth of the filter, therefore the disturbance rejection capability of the filter deteriorates. As can be understood in Figure 5b and Figure 6b, increasing in $k_{2}$ reduces the overshoot; however, it causes the dynamic response of the filter to slow down. In order to provide a trade-off between response time and filtering capability, the gains $k_{1}$ and $k_{2}$ are preferred as 2.82 and 0.25, respectively.

It can be observed in Figure 4, the DC offset rejection loop in the FOGI estimates the DC offset component of input currents ($i_{DC}$) using an additional integrator with a gain of $k_{1}\hat{\omega }$. Next, $i_{\mathrm{DC}}$ is added to $i_{L\alpha }^{\prime }$ and $i_{L\beta }^{\prime }$ to obtain *i_DC_fb_*, which is then subtracted from the input currents $i_{L\alpha }$ and $i_{L\beta }$ to reject the DC offset component. The transfer functions R(s) in Equation (1) and Q(s) in Equation (2) verify this situation. As can be seen, the DC offset rejection loop adds a zero to the origin of both Equations (1) and (2). It means that it provides a zero gain at 0 Hz, and thus, totally eliminates the DC offset component.

As shown in Figure 3, the filtered load currents at FOGI outputs (i.e., $i_{L\alpha }^{\prime }$, $qi_{L\alpha }^{\prime }$, $i_{L\beta }^{\prime }$, and $qi_{L\beta }^{\prime }$) are applied to the positive sequence calculator (PSC) unit to achieve the fundamental frequency positive sequence (FFPS) components of the load currents. To extract the FFPS components ($i_{L\alpha }^{+}$ and $i_{L\beta }^{+}$), instantaneous symmetrical components theory is realized as [37]:

(3) $$\left[\begin{array}{c} i_{L\alpha }^{+}\\ i_{L\beta }^{+} \end{array}\right]=\frac{1}{2}\left[\begin{array}{cc} 1 & -q\\ q & 1 \end{array}\right]\left[\begin{array}{c} i_{L\alpha }^{\prime }\\ i_{L\beta }^{\prime } \end{array}\right]$$

After that, the FFPS components are passed through a quasi-type-1 PLL (QT1-PLL) [38] to determine the frequency (f) and amplitude ($I_{Lm}$) of the load currents. QT1-PLL offers a fast transient response and great harmonic rejection capability at the fundamental frequency. However, it suffers from errors in amplitude estimation in case of frequency drifts.

To overcome this problem, an amplitude detection mechanism (ADM) unit is added to the QT1-PLL structure, as observed from Figure 3. This ensures that the amplitude detection remains accurate even if the frequency changes. ADM is expressed as:

(4) $$I_{Lm}=\sqrt{{\bar{i}}_{Ld}^{2}+{\bar{i}}_{Lq}^{2}}$$

In QT1-PLL, two moving average filters (MAF) are employed that can act as an ideal low-pass filter. The transfer function of MAF in *s*-domain can be obtained as [39]:

(5) $$\mathrm{MAF}\left(s\right)=\frac{1-e^{-T_{\omega }s}}{T_{\omega }s}$$

where $T_{\omega }$ signifies its window-length. To implement the MAF for digital applications, its transfer function in z-domain can be expressed as [29,30,31,32,33,34]:

(6) $$\mathrm{MAF}\left(z\right)=\frac{1}{N}\frac{1-z^{-N}}{1-z^{-1}}$$

where N is the order of the MAF and equals $f_{s}T_{\omega }$ (here, $f_{s}$ represents the sampling frequency).

**Figure 7:**
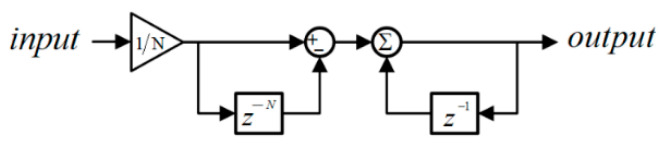
Block structure of MAF in the z-domain.

Based on Equation (6), the block structure of the MAF can be easily derived, as illustrated in Figure 7. The MAF’s order N should be set the proper value to block the harmonics in the load currents. In SEIG-based systems, imbalances caused by unbalanced loads and DC offsets caused by measurement faults lead to serious problems. Fortunately, imbalances and DC offsets are removed by means of FOGI filters before the QT1-PLL. The FOGIs also suppressed the high-frequency harmonic components effectively because it exhibits a band-pass filter feature. But the FOGI filters cannot adequately filter low-order harmonic components, especially the −5th- and +7th-order harmonics. These low-order harmonics caused by nonlinear loads are the most dominant components in the three-phase applications [23]. Therefore, to block the harmonics orders of −5 and +7, the window-length $T_{\omega }$ is selected as T/3 in this study (where, T is the period of the load currents). Considering $T_{\omega }=T/3$ and $f_{s}=5\;\mathrm{kHz}$, MAF’s order N is calculated by $f_{s}T_{\omega }$ (here, $f_{s}$ represents the sampling frequency), results in 33 for the 50 Hz system.

### 3.2. Parameter Design and Stability Analysis of ADFOGI-PLL

As the gains $k_{1}$ and $k_{2}$ in FOGI and the MAF’s order N are obtained in Section 3.1, the proportional gain $k_{p}$ in the control loop of QT1-PLL shown in Figure 3 should be set properly for ensuring the stability of the system.

**Figure 8:**
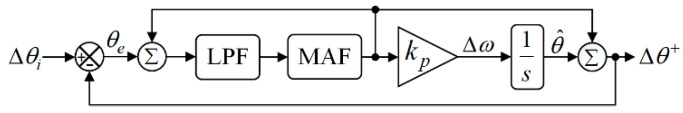
Small-signal model of proposed ADFOGI-PLL.

In this context, the small-signal model of proposed ADFOGI-PLL is commonly used. Based on [18,21], its small-signal model can be easily derived, as illustrated in Figure 8. In this model, FOGIs are considered as a first-order low-pass filter (LPF) in the low-frequency range, as discussed in detail in [18]. The first-order LPF can be expressed as:

(7) $$\mathrm{LPF}\left(s\right)=\frac{\omega_{p}}{s+\omega_{p}}$$

where $\omega_{p}$ denotes the cut-off frequency. In this study, $\omega_{p}$ is set to 2π22 rad/s based on [40].

Considering the small-signal model given in Figure 8, the open-loop transfer function of ADFOGI-PLL can be obtained as:

(8) $$G_{ol}(s)=\frac{\Delta \theta^{+}(s)}{\theta_{e}}=\left(\frac{\mathrm{LPF}(s)\mathrm{MAF}(s)}{1-\mathrm{LPF}(s)\mathrm{MAF}(s)}\right)\left(1+\frac{k_{p}}{s}\right)$$

**Figure 9:**
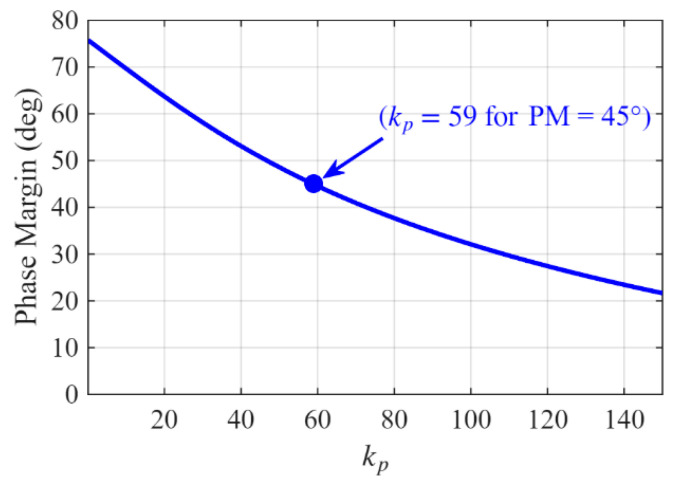
PM variations as a function of $k_{p}$.

Based on Equation (8), phase margin (PM) variations as a function of $k_{p}$ can be determined as shown in Figure 9. In the literature, to ensure the stability of the system, the PM within the range of 30–60° is recommended [23,38]. In this paper, the PM of the proposed ADFOGI-PLL is selected as 45°, which is the midpoint of this range, corresponding to $k_{p}$ = 59. Figure 9 also shows that the proposed ADFOGI-PLL ensures the stability of the system.

### 3.3. Performance Comparison of Proposed ADFOGI-PLL

**Figure 10:**
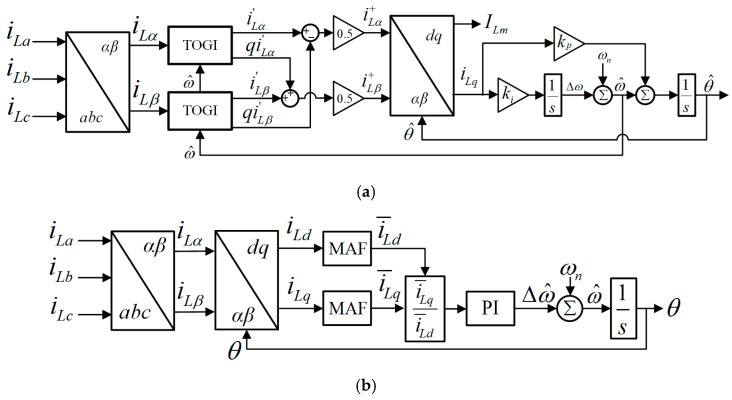
Block diagram of two enhanced PLL methods: (**a**) DTOGI-PLL, (**b**) MAF-PLL.

The effectiveness of the proposed ADFOGI-PLL should be evaluated before using it in DSTATCOM control. For this reason, to verify its performance, it is experimentally compared with two well-known enhanced PLL methods under several test conditions. These methods are DTOGI-PLL [36] and MAF-PLL [39], as shown in Figure 10. For the sake of readability, the details of these two PLL methods are not included in this study. However, detailed information about the DTOGI-PLL and MAF-PLL can be found in [18,36,39].

The experimental performance validation of the MAF PLL, DTOGI PLL, and proposed ADFOGI PLL methods was performed using the TMS320F28335 digital signal processor (DSP). In the experimental studies, the sampling frequency, fundamental network angular frequency, and voltage amplitude were set to 10 kHz, 2π × 50 rad/s, and 1 unit (p.u.), respectively.

In the experimental studies conducted using a digital signal processor (DSP), the amplitudes of the input signals and the sampling frequency are considered as 1 p.u. and 5 kHz, respectively. The control parameters of all PLL methods are provided in Table 2.

Two test cases are considered:

Case-1: To test the DC offset immunity of the methods, a DC offset component is generated in the load currents of phase-A. In this case, this component is set to 0.1 p.u. In the presence of DC offset, the frequency is also dropped from 50 Hz to 47 Hz.

Case-2: To represent unbalanced and nonlinear load conditions, both imbalances and harmonics are created in the load currents. For imbalances, the amplitudes of the load currents ($i_{La}$, $i_{Lb}$, and $i_{Lc}$) are adjusted to 1 p.u., 1.2 p.u., and 0.8 p.u., respectively, while for harmonics, 5th- and 7th-order harmonics are added to each load current (where $V_{5}^{-}=0.04∠-90{}^{\circ}$ and $V_{7}^{+}=0.04∠0{}^{\circ}$ in accordance with IEEE Std. 1547-2018 [41]). Additionally, in this case, the frequency is stepped up from 50 Hz to 52 Hz to test the response of the PLLs to frequency changes.

**Figure 11:**
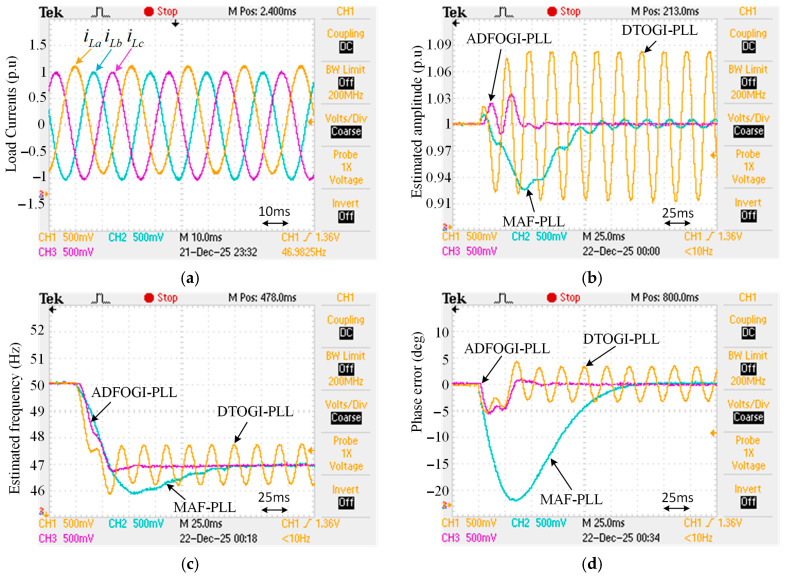
Case-1: DC offset condition. (**a**) Load currents, (**b**) estimated amplitude, (**c**) estimated frequency, and (**d**) phase error.

Figure 11 shows the experimental results of Case-1 in the presence of DC offset component. As can be seen, the proposed ADFOGI-PLL offers superior performance over DTOGI-PLL and MAF-PLL in terms of accuracy, dynamic response, and overshoot, even if the frequency drifts. For example, the settling-time of MAF-PLL is 149 ms, while the settling time of ADFOGI-PLL is only 38 ms. Although the dynamic response of MAF-PLL is too slow, it provides satisfactory accuracy in amplitude, frequency, and phase estimation. However, DTOGI-PLL produces significant errors, making it difficult to use in DSTATCOM control.

The numerical results of this test are provided in detail in Table 3. As a result, it is confirmed that the proposed PLL method demonstrates excellent performance compared to the other two methods in the presence of the DC offset.

**Figure 12:**
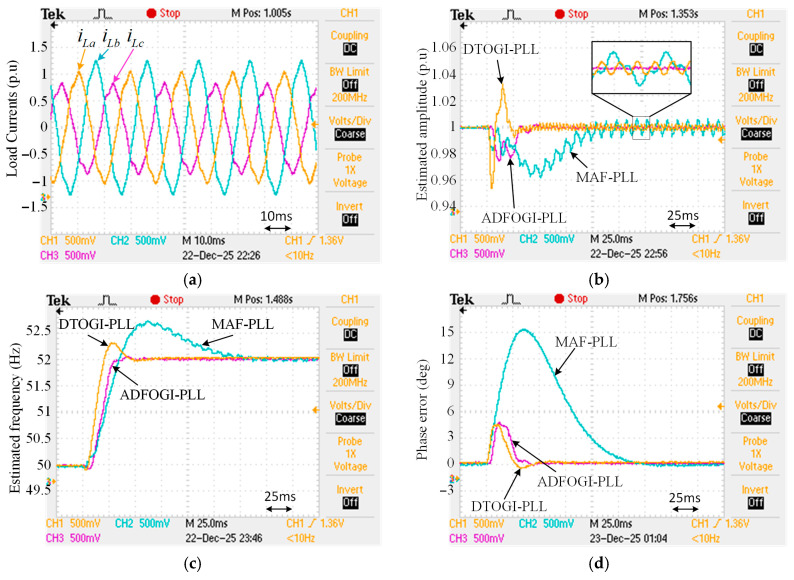
Case-2: imbalances and harmonics condition. (**a**) Load currents, (**b**) estimated amplitude, (**c**) estimated frequency, and (**d**) phase error.

Figure 12 depicts the experimental results of Case-2 under heavily distorted load currents. As observed, ADFOGI-PLL provides the best performance in the transient state and steady state. That is, it presents the least settling time and the lowest overshoots in amplitude, frequency, and phase detection. Detailed results are presented in Table 3. The performance of DTOGI-PLL is satisfactory, while the MAF-PLL causes errors in estimating the load currents information, especially in amplitude estimation, due to the frequency variations, this can negatively affect the DSTATCOM control. Consequently, this test demonstrates the effectiveness of the proposed ADFOGI-PLL under nonlinear and unbalanced load conditions.

### 3.4. SEIG Voltage Magnitude Estimation and Unit Vector Generation

As depicted in Figure 3, the control algorithm requires both the SEIG voltage magnitude and the unit voltage templates (UVTs) to derive the reference source currents $\left(i_{sa}^{*},\;i_{sb,}^{*}i_{sc}^{*}\right)$ Additionally, the in-phase UVTs together with their quadrature counterparts are produced to ensure proper synchronization between the DSTATCOM output voltages and the SEIG terminal voltages.

As detailed in Equation (9), the three-phase SEIG voltages are calculated using the line-to-line voltages ($v_{sab}$ and $v_{sbc}$) obtained from two voltage sensors.

(9) $$\begin{array}{l} v_{sa}=\frac{2v_{sab}+v_{sbc}}{3}\\ v_{sb}=\frac{-v_{sab}+v_{sbc}}{3}\\ v_{sc}=\frac{-v_{sab}-2v_{sbc}}{3} \end{array}$$

Using Equation (10), the peak value of the SEIG voltages is determined.

(10) $$V_{t}=\sqrt{\frac{2}{3}(v_{sa}^{2}+v_{sb}^{2}+v_{sc}^{2})}$$

Using Equation (11), the in-phase UVTs are obtained.

(11) $$u_{pa}=\frac{v_{ab}}{V_{t}};\;u_{pb}=\frac{v_{bc}}{V_{t}};\;u_{pc}=\frac{v_{ca}}{V_{t}}$$

The quadrature UVTs are then calculated as in Equation (12).

(12) $$\begin{array}{l} u_{qa}=\frac{-u_{pb}+u_{pc}}{\sqrt{3}}\\ u_{qb}=\frac{3u_{pa}+u_{pb}-u_{pc}}{2\sqrt{3}}\\ u_{qc}=\frac{-3u_{qa}+u_{qb}-u_{qc}}{2\sqrt{3}} \end{array}$$

The magnitudes of the fundamental active component ($I_{Lap}$) and reactive component ($I_{Laq}$) of the load currents are estimated from the magnitude of the load currents ($I_{Lm}$), the unit magnitude voltage vectors ($u_{pa}$ and $u_{qa}$), the zero crossing detector (ZCD) and the sample and hold (S&H) as shown in Figure 2. The other active and reactive components are calculated in a similar way.

### 3.5. Fundamental Active Current Components Estimation

To ensure that the SEIG terminal voltages remain at the desired frequency of 50 Hz, it is crucial to extract the active current components accurately. For this purpose, the frequency estimated by the proposed ADFOGI-based algorithm is compared with its reference value, and the resulting frequency deviation is regulated by a PI controller. The PI controller’s output ($I_{fp}$) represents the active current component that must be compensated. The fundamental average active current component ($I_{Lpavg}$) is obtained according to Equation (13). Subsequently, the magnitude of the active component of the reference current ($I_{spt}$) is determined by subtracting $I_{fp}$ from the $I_{Lpavg}$ expressed in Equation (14).

(13) $$I_{Lpavg}=\frac{I_{Lap}+I_{Lbp}+I_{Lcp}}{3}$$

(14) $$I_{spt}=I_{Lpavg}-I_{fp}$$

The reference active component of each phase is calculated by multiplying the corresponding phase’s in-phase unit templates by the $I_{spt}$ value, as demonstrated in Equation (15).

(15) $$i_{sap}^{*}=I_{spt}u_{pa};\;i_{sbp}^{*}=I_{spt}u_{pb};\;i_{scp}^{*}=I_{spt}u_{pc}$$

### 3.6. Fundamental Reactive Current Components Estimation

To regulate the peak magnitude of the SEIG terminal voltages at the desired level, it is necessary to extract the corresponding reactive current components. For this purpose, the error between the measured SEIG voltage amplitude ($V_{t}$).) and its reference value ($V_{tref}$) is fed into a PI controller. The output of the PI controller corresponds to the reactive current component that must be compensated ($I_{vtq}$). The fundamental average reactive current component ($I_{Lqavg}$) is then obtained using Equation (16). Finally, the reactive component of the reference current ($I_{sqt}$) is derived by subtracting $I_{Lqavg}$ from $I_{vtq}$, as presented in Equation (17).

(16) $$I_{Lqavg}=\frac{I_{Laq}+I_{Lbq}+I_{Lcq}}{3}$$

(17) $$I_{sqt}=I_{vtq}-I_{Lqavg}$$

The reference active current component of each phase is obtained by multiplying the quadrature unit voltage templates by the value of $I_{spt}$, as expressed below.

(18) $$i_{saq}^{*}=I_{sqt}u_{qa};\;i_{sbq}^{*}=I_{sqt}u_{qb};\;i_{scq}^{*}=I_{sqt}u_{qc}$$

### 3.7. Reference Current Estimation

Based on Equations (15) and (18), the reference source currents ($i_{sa}^{*}$, $i_{sb}^{*}$, and $i_{sc}^{*}$) generated at the output of the RCC block, shown in Figure 3, are expressed as:

(19) $$i_{sa}^{*}=i_{sap}^{*}+i_{saq}^{*};\;i_{sb}^{*}=i_{sbp}^{*}+i_{sbq}^{*};\;i_{sc}^{*}=i_{scp}^{*}+i_{scq}^{*}$$

In this study, a current control strategy based on sinusoidal pulse width modulation (SPWM) is employed. The reference currents obtained from Equation (19) are then compared with the SEIG current measured during the switching signal generation (SSG) stage; the resulting sinusoidal current error is compared with a 10 kHz carrier triangular wave. As a result, as shown in Figure 3, SPWM switching pulses (S_1_, S_2_, …, S_6_) at a 10 kHz frequency are generated at the output of the SSG unit. These switching pulses are then applied to the IGBT switches of the VSC shown in Figure 1.

## 4. Results and Discussion

**Figure 13:**
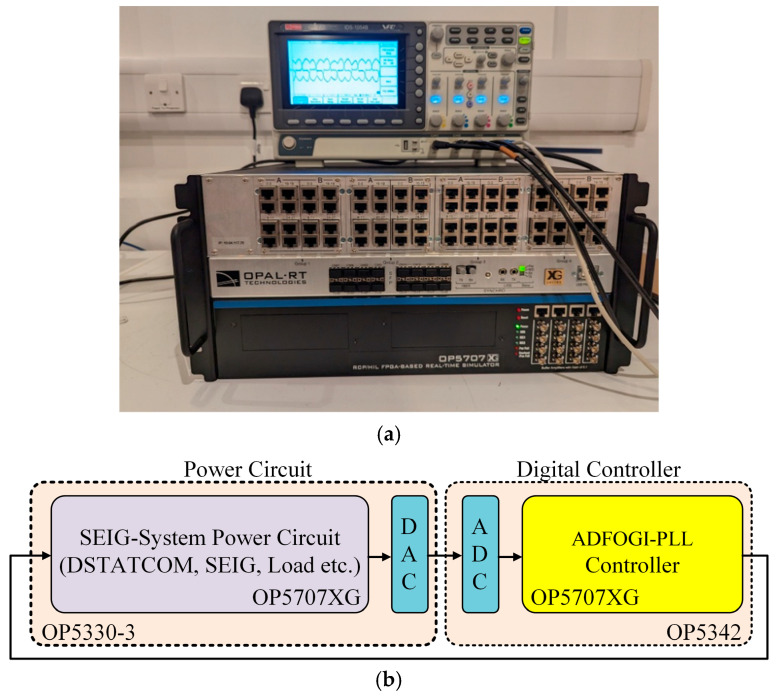
Real-time experimental setup: (**a**) setup based on OPAL-RT system, (**b**) block diagram of configuration.

This section investigates the performance of the proposed ADFOGI-based DSTATCOM control strategy using an OPAL-RT real-time execution platform. OPAL-RT is a widely validated digital system known for its ability to accurately emulate physical power systems and perform hardware-in-the-loop (HIL) tests under true real-time operating conditions [42,43,44]. Through this platform, detailed models of power systems, microgrids, motor drive structures, and renewable energy units can be executed under real-time conditions. Figure 13a presents the experimental setup constructed on the OPAL-RT platform.

In this work, both the power stage (SEIG, DSTATCOM, etc.) and the proposed control algorithm are executed in real time on the OP5707XG simulator (OPAL-RT Technologies, Montreal, QC, Canada), operating with a sampling rate of 20 kHz. The switching frequency of the voltage source converter (VSC) is set to 10 kHz, consistent with the SPWM-based current control strategy. The load currents, DSTATCOM currents and SEIG voltage, and current signals are converted into analog signals through the OP5330-3 digital-to-analog converter (DAC) module (OPAL-RT Technologies, Montreal, QC, Canada). These analog signals are then sampled at a rate of 20 kHz by the OP5342 analog-to-digital converter (ADC) module (OPAL-RT Technologies, Montreal, QC, Canada) for use in the controller. A detailed block schematic of the configuration is provided on Figure 13b. The corresponding experimental waveforms were captured and visualized using a Rohde & Schwarz RTM3004 digital storage oscilloscope (Rohde & Schwarz GmbH and Co. KG, Munich, Germany) with four channels, 350 MHz bandwidth, 2.5 GSPS sampling rate, 40 Mpts memory, and 1 ns resolution.

### 4.1. Performance of SEIG–DSTATCOM System Under the Nonlinear Load with DC Offset Conditions

Modern power systems frequently encounter nonlinear loads, which cause the generation of harmonic components. To evaluate the performance of the proposed ADFOGI-based control structure under nonlinear load conditions, a three-phase diode rectifier DC load is connected to the PCC of the system. In this test DTOGI scenario, the nonlinear load is kept constant, and the performance of the control algorithm against DC offset effects is also investigated. In real systems, DC components may occur in phase currents due to magnetic saturation, offset drift, or ADC-based measurement errors associated with sensors. To represent this situation, a DC offset of +3 A is added to phase a, +6 A to phase b, and −5 A to phase c, and the behavior of the load currents (*i_Labc_*) is observed.

**Figure 14:**
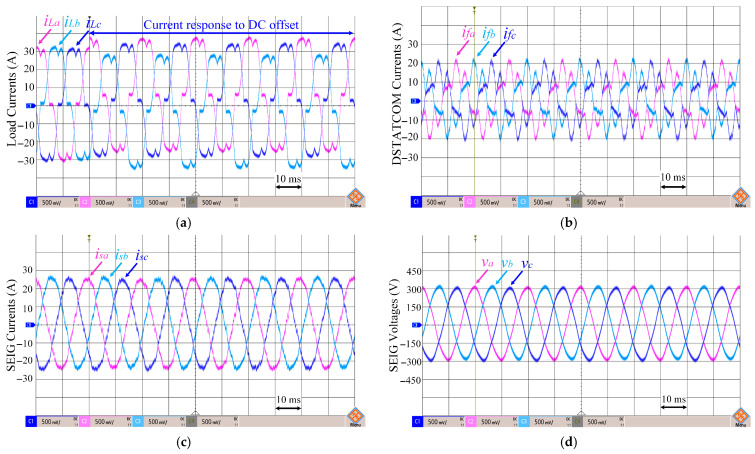
Waveforms under nonlinear load with DC offset: (**a**) load currents, (**b**) DSTATCOM currents, (**c**) SEIG currents, (**d**) SEIG terminal voltages.

In Figure 14, the load currents (*i_Labc_*), DSTATCOM currents (*i_fabc_*), SEIG currents (*i_sabc_*), and SEIG terminal voltages (*v_ab_*, *v_bc_*, *v_ca_*) for the proposed ADFOGI-based control algorithms are shown under a nonlinear load.

**Figure 15:**
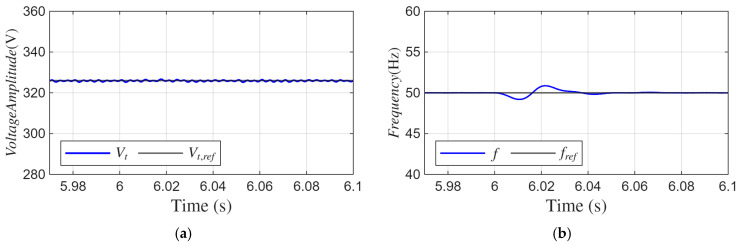
Waveforms under nonlinear load with DC offset: (**a**) amplitude of SEIG voltages, (**b**) SEIG frequency.

Figure 15 illustrates the amplitude of the SEIG voltages (*V_t_*) and the SEIG frequency (*f*) under nonlinear load. The structure of the nonlinear load causes the load currents (*i_Labc_*) to contain high harmonics (16.25% THD), and a shift in the load currents occurs after applying the DC offset, as shown in Figure 14a. The proposed ADFOGI-based control algorithm correctly separates the deviations caused by DC offset, extracts only the fundamental component, and generates the reference current accordingly. As a result, it can be seen that DSTATCOM successfully injects the necessary currents (*i_fabc_*) (Figure 14b) to suppress both harmonic components and changes caused by the DC offset.

Thanks to these injected currents, the SEIG currents (Figure 14c) remain balanced and symmetrical, close to the fundamental component, despite harmonic currents and DC offset distortions. Similarly, the SEIG phase voltages (Figure 14d) maintain their sinusoidal and stable structure, independent of DC offset effects.

Total harmonic distortion (THD) values obtained also confirm these results. As shown in Table 4, under nonlinear load with DC offset, the THD of the load current has been measured at 16.23%, while after compensation, the THD of the SEIG current has decreased to 3.71%, and the SEIG phase voltage THD has decreased to 1.66%. Thanks to the proposed ADFOGI-based control method, the THD values remain within the IEEE-519-2022 standard limits.

Figure 15 illustrates the peak value of the SEIG terminal voltage and the frequency dynamic response of the DSTATCOM-based SEIG system under nonlinear load conditions with DC offset. As shown in Figure 15a, the SEIG voltage peak value remains close to the reference level of 326.5 V, with a maximum deviation of less than 2 V, and no noticeable steady-state oscillation is observed.

As presented in Figure 15b, the frequency exhibits a maximum undershoot of approximately 0.8 Hz and a maximum overshoot of approximately 0.9 Hz relative to the 50 Hz reference. The frequency settles back to its nominal value within approximately 20–25 ms, indicating a fast dynamic response and stable operation under disturbance conditions.

### 4.2. Performance of SEIG–DSTATCOM System Under the Unbalanced and Nonlinear Load with DC Offset Conditions

In modern power systems, unbalanced load conditions that affect system performance are a common occurrence. Unbalanced loads typically arise from devices with different load values being connected to the same system. Additionally, DC offset originating from measurement errors may be present in the system.

To test the proposed ADFOGI-based control system under these conditions, an unbalanced load consisting of resistors with values of 20 Ω, 35 Ω, and 15 Ω is connected to the PCC in addition to the nonlinear load. Furthermore, a DC offset of +3 A was added to phase a, +6 A to phase b, and −5 A to phase c. To test its performance during load variations, the load is increased by connecting a second load with the same values in parallel. These results demonstrate that the proposed ADFOGI control structure can effectively maintain voltage and frequency stability not only under harmonic conditions but also under measurement-induced DC offset effects.

**Figure 16:**
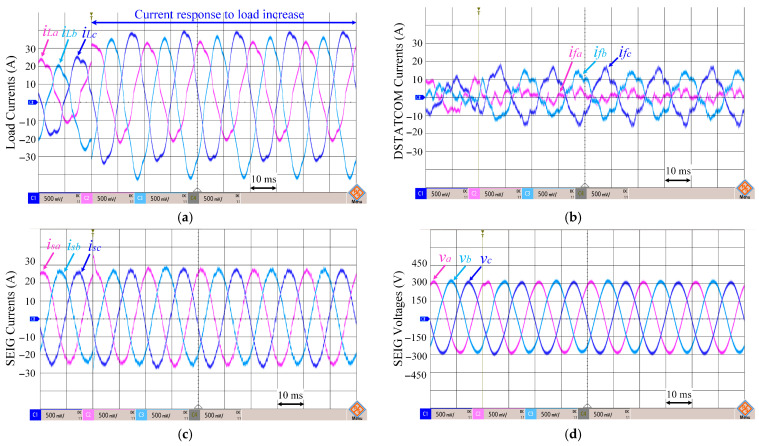
Waveforms under unbalanced and nonlinear load with DC offset: (**a**) load currents, (**b**) DSTATCOM currents, (**c**) SEIG currents, (**d**) SEIG terminal voltages.

**Figure 17:**
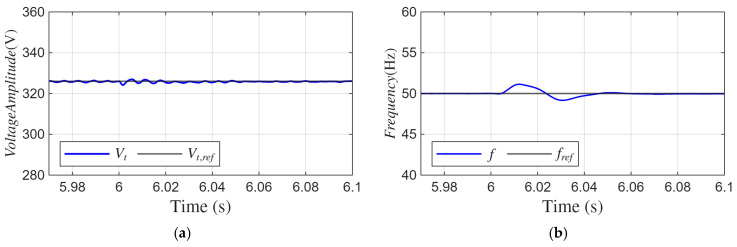
Waveforms under unbalanced and nonlinear load with DC offset: (**a**) amplitude of SEIG voltages, (**b**) SEIG frequency.

In Figure 16, the load currents (*i_Labc_*), DSTATCOM currents (*i_fabc_*), SEIG currents (*i_sabc_*), and SEIG terminal voltages (*v_ab_*, *v_bc_*, *v_ca_*) for the proposed ADFOGI-based control algorithm are shown under unbalanced and nonlinear load with DC offset. Figure 17 illustrates the amplitude (*V_t_*) and frequency (*f*) of the SEIG voltages.

The currents drawn by the unbalanced and nonlinear load (*i_Labc_*) contain harmonics and create significant imbalance between phases, as shown in Figure 16a. Additionally, DC offset components are present in the load currents. When the second load is connected, the current drawn by the load increases, and the imbalance between phases becomes more pronounced. The proposed ADFOGI-based control algorithm accurately calculates the required compensation currents for DSTATCOM.

As shown in Figure 16b, the DSTATCOM injects higher amplitude currents to meet the reactive power demand that increased with the load increase and to effectively suppress the imbalance.

As a result, SEIG output currents (Figure 16c) remain balanced and close to the fundamental component despite the load containing imbalance, harmonics, and DC offset. SEIG phase voltages (Figure 16d) also maintain their sinusoidal structure despite these challenging test conditions.

Under unbalanced and nonlinear load and DC offset error, the load current THD is measured at 9.12%, while the SEIG current THD after compensation is 1.97%, and the SEIG phase voltage THD is only 0.56%. As seen, the results obtained are well below the IEEE-519-2022 standard values.

The peak value (*V_t_*) of the SEIG terminal voltage shown in Figure 17a remains around the reference value of 326.5 V under DC offset, unbalanced and nonlinear load conditions. During the transient interval, the maximum deviation is approximately ±2 V, and the voltage quickly returns to the reference level without steady-state oscillation.

Figure 17b shows the frequency response. When the load increase occurs, the frequency exhibits an overshoot of approximately 1 Hz relative to the 50 Hz reference. The frequency returns to 50 Hz within nearly two fundamental periods (about 40 ms). No sustained oscillation is observed after the transient.

These results show that the proposed ADFOGI-based control method maintains the voltage amplitude and frequency stability with limited transient deviation under severe operating conditions.

### 4.3. Performance of SEIG–DSTATCOM System Under the One-Phase Open-Circuit (OPOC) Fault with DC Offset Test Condition

In three-phase systems, under certain fault or load conditions, it is possible for one of the phases to remain open-circuited. To test this fault condition, the performance of the SEIG–DSTATCOM control method is evaluated under unbalanced conditions where one phase of the load/line is open-circuited, and current is drawn from the other two phases. Additionally, to evaluate the control structure’s robustness to measurement errors, DC offset components are applied to the phase currents. In this context, a DC offset of +3 A is added to phase a, +6 A to phase b, and −5 A to phase c.

**Figure 18:**
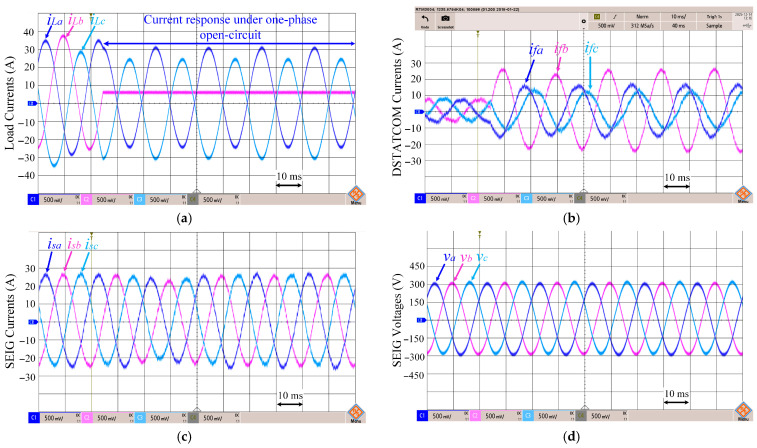
Waveforms under one-phase open-circuit fault with DC offset: (**a**) load currents, (**b**) DSTATCOM currents, (**c**) SEIG currents, (**d**) SEIG terminal voltages.

**Figure 19:**
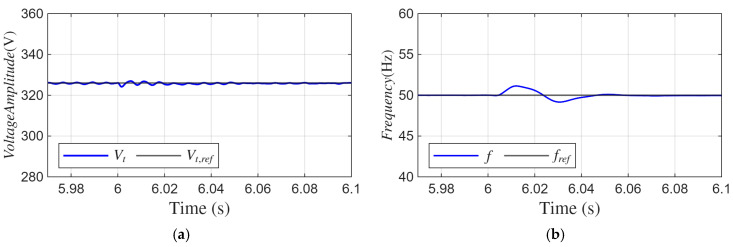
Waveforms under one-phase open-circuit fault with DC offset: (**a**) amplitude of SEIG voltages, (**b**) SEIG frequency.

In Figure 18, the load currents (*i_Labc_*), DSTATCOM currents (*i_fabc_*), SEIG currents (*i_sabc_*), and SEIG terminal voltages (*v_ab_*, *v_bc_*, *v_ca_*) of system for proposed ADFOGI-based control algorithm are shown under the OPOC condition. Figure 19 illustrates the amplitude (*V_t_*) and frequency (*f*) of the SEIG voltages under the OPOC conditions.

When the SEIG supplies a balanced three-phase load, phase B of the load is disconnected, so theoretically, the *i_Lb_* current should be zero. However, due to DC offset components added to the phase currents, the phase B current appears as a small current shift at the DC level in the measurement, even though no current flows to the load (Figure 18a). Similarly, current shifts due to DC offset are also present in the A and C phases.

Even under these challenging operating conditions, the proposed ADFOGI control algorithm correctly generates the reference currents and enables the DSTATCOM to produce the currents required for the system (Figure 18b). Thus, the currents drawn from the SEIG remain balanced and maintain their sinusoidal structure (Figure 18c). Since the SEIG currents remain balanced as a result of DSTATCOM compensation, the SEIG phase voltages also continue symmetrically and stably (Figure 18d).

As shown in Figure 19a, when one phase is opened, the peak value of the SEIG terminal voltage remains close to the reference value of 326.5 V. The maximum transient deviation is limited to approximately ±3 V, and no steady-state oscillation is observed after the disturbance.

Figure 19b presents the frequency response. When the single phase is disconnected, the frequency exhibits a transient deviation of approximately 3.4 Hz relative to the 50 Hz reference. The frequency settles back to 50 Hz within nearly three periods. The results obtained indicate that the proposed ADFOGI-based control method maintains stable voltage amplitude and frequency regulation even under one-phase open-circuit conditions.

### 4.4. Comparison of Proposed Method and Key Findings

To demonstrate the performance of the proposed method, a comparison is presented with existing synchronization techniques found in the literature.

Table 4 presents a quantitative comparison between the proposed ADFOGI-PLL-based control method and existing approaches reported in [10,45], and [46]. Under all three challenging operating conditions, the proposed method achieves lower SEIG current and SEIG voltage THD values compared to the Enhanced Phase-Locked Loop–Current Synchronous Detection (EPLL-CSD), SOGI-CSD, and SRF-PLL-based methods.

Under nonlinear load conditions with DC offset, the proposed method reduces SEIG current THD to 3.71%, while the compared methods produce values between 4.30% and 4.76%, corresponding to approximately 22% improvement over the SRF-PLL-based approach. Under unbalanced and nonlinear conditions, SEIG current THD decreases to 1.97%, providing nearly 57% improvement compared to conventional synchronization techniques. In addition, under one-phase open-circuit conditions, the improvement exceeds 59%. These consistent reductions in distortion levels confirm the improved harmonic suppression capability, robustness, and practical applicability of the proposed ADFOGI-based control method.

In all scenarios, SEIG current and SEIG voltage THD values remain at low levels, indicating effective harmonic suppression and stable synchronization performance. According to IEEE 519-2022, the recommended voltage THD limit at the point of common coupling (PCC) is 5%. In all test scenarios, the proposed method maintains the SEIG voltage THD below 2%, providing a clear safety margin relative to the standard threshold.

Moreover, the low SEIG current distortion levels after compensation confirm the effectiveness of the proposed synchronization and harmonic filtering mechanism, even under severe nonlinear load conditions.

From an application perspective, the results indicate that the proposed control method is suitable for isolated wind energy systems exposed to harmonic distortion, load imbalance, and measurement-induced DC offset.

## 5. Conclusions

In this study, an Advanced Dual Fourth-Order Generalized Integrator (ADFOGI)-based DSTATCOM control method is proposed to enhance voltage and frequency stability and improve power quality in SEIG-based isolated wind power systems.

The performance of the proposed algorithm has been evaluated on the OPAL-RT real-time platform under three challenging operating conditions: (i) nonlinear load with DC offset, (ii) nonlinear and unbalanced load conditions with DC offset (including dynamic load increase), and (iii) one-phase open-circuit fault in the presence of DC offset.

Despite these comprehensive tests, the proposed ADFOGI method has successfully suppressed DC offset components in all scenarios, effectively reduced harmonic and imbalance-induced distortions. As a result, THDs of SEIG voltages and SEIG currents meet the IEEE-519-2022 standards under all conditions. Furthermore, the system has continued to maintain voltage/frequency stability even under severe operating conditions such as high harmonic content, load imbalance, and open-circuit faults.

The results obtained demonstrate that the proposed ADFOGI-based control algorithm exhibits high resilience against measurement errors, harmonic distortion, and imbalance conditions that may be encountered in real systems. In this respect, ADFOGI offers a reliable, effective, and practical solution for both voltage/frequency regulation and power quality improvement in SEIG-based isolated power systems.

### Limitations and Future Work

The performance of the ADFOGI architecture is sensitive to parameter settings, and adjusting these parameters under different operating conditions directly affects system performance. As shown in Figure 9, when the phase margin is 60, $k_{p}$ = 27, and when the phase margin is 30, $k_{p}$ = 108. This indicates that the system is stable within the $k_{p}$ value range of 27 to 108 and defines the system’s stability boundary.

In future work, researchers may be advised to focus on a control algorithm incorporating lower-order filter structures in order to achieve the superior performance of the proposed ADFOGI structure against sensor-induced errors.

## Figures and Tables

**Table 1:** Parameters.

**SEIG Parameters**	
Power	15 kW
Voltage (L-L)	400 V
Stator resistance	0.2147 Ω
Stator inductance	0.000991 H
Rotor resistance	0.2205 Ω
Rotor inductance	0.000991 H
Mutual inductance	0.06419 H
Frequency	50 Hz
Pole pairs	2
Excitation capacitor bank	C_Y_ = 270 μF (Y-connected)
**DSTATCOM Parameters**	
Interfacing inductor (Lf)	5 mH
DC bus capacitor (Cdc)	2500 μF
Switching frequency	10 kHz

**Table 2:** Control parameters of all PLLs.

Parameters	DTOGI-PLL	MAF-PLL	Proposed ADFOGI-PLL
Proportional gain, $k_{p}$	220	41.67	59
Integral gain, $k_{i}$	20167	723.38	-
Gains, $k_{1}-k_{2}$	2.82 – 1.1	-	2.82 – 0.25
MAF’s window-length, $T_{\omega }$	-	T	T/3
MAF’s order, N		100	33

**Table 3:** Experimental results of PLLs.

	DTOGI-PLL	MAF-PLL	ADFOGI-PLL
Case-1: DC offset			
2% settling time	-	149 ms (7.45 cycles)	38 ms (1.9 cycles)
Peak-to-peak amplitude error	0.18 p.u.	0.009 p.u.	0 p.u.
Frequency overshoot	1.1 Hz	1.07 Hz	0.074 Hz
Phase overshoot	5°	22.2°	5.4°
Case-2: Imbalances and harmonics			
2% settling time	42 ms (2.1 cycles)	168 ms (8.4 cycles)	36.5 ms (1.8 cycles)
Peak-to-peak amplitude error	0.005 p.u.	0.013 p.u.	≈0 p.u.
Frequency overshoot	0.3 Hz	0.7 Hz	≈0 Hz
Phase overshoot	4.3°	15°	4.6°

**Table 4:** Comparison of proposed method with existing methods.

	Proposed ADFOGI-PLL Based Control Method			EPLL-CSD Theory [10]			SOGI-CSD Theory [45]			SRF-PLL Based *dq*-Theory [46]		
Test Conditions	Load Current THD	SEIG Current THD	SEIG Voltage THD	Load Current THD	SEIG Current THD	SEIG Voltage THD	Load Current THD	SEIG Current THD	SEIG Voltage THD	Load Current THD	SEIG Current THD	SEIG Voltage THD
Nonlinear load with DC Offset	16.23%	3.71%	1.66%	16.15%	4.21%	1.92%	16.55%	4.30%	2.15%	17.12%	4.76%	2.46%
Unbalanced and nonlinear load with DC Offset	9.12%	1.97%	1.92%	9.25%	2.53%	2.49%	9.82%	3.25%	2.60%	9.95%	4.56%	2.72%
One-phase open-circuit with DC Offset	1.66%	1.92%	0.74%	1.75%	4.37%	1.99%	1.80%	4.55%	2.89%	1.89%	4.70%	3.89%

## Data Availability

The original contributions presented in this study are included in the article. Further inquiries can be directed to the corresponding author.

## Abbreviations

The following abbreviations are used in this manuscript:

ADFOGI	Advanced Dual Fourth-Order Generalized Integrator
ALES-PLL	Adaptive Least-Error Squares Phase-Locked Loop
AANF	Amplitude Adaptive Notch Filter
ANN	Artificial Neural Network
BESS	Battery Energy Storage System
CSD	Current Synchronous Detection
DC	Direct Current
DSTATCOM	Distribution Static Compensator
DSC	Delayed Signal Cancellation
DSOGI-PLL	Dual Second-Order Generalized Integrator Phase-Locked Loop
DVR	Dynamic Voltage Restorer
EPLL	Enhanced Phase-Locked Loop
FFPS	Fundamental Frequency Positive Sequence
FOGI	Fourth-Order Generalized Integrator
GA-FLC	Genetic Algorithm-Based Fuzzy Logic Controller
LMS	Least Mean Square
LMF	Least Mean Fourth
LPF	Low-Pass Filter
MAF	Moving Average Filter
MLMS	Momentum Least Mean Square
MSOGI	Multiple Second-Order Generalized Integrator
PCC	Point of Common Coupling
PM	Phase Margin
PMSG	Permanent Magnet Synchronous Generator
PSC	Positive Sequence Calculator
PWM	Pulse Width Modulation
QNLMF	Quasi-Newton Least Mean Fourth
QT1-PLL	Quasi-Type-1 Phase-Locked Loop
SEIG	Self-Excited Induction Generator
SOGI-FLL	Second-Order Generalized Integrator Frequency-Locked Loop
SRF-PLL	Synchronous Reference Frame Phase-Locked Loop
SVC	Static Var Compensator
S&H	Sample and Hold
THD	Total Harmonic Distortion
UVT	Unit Voltage Template
ZCD	Zero Crossing Detection

## Appendix A

This section presents the calculation of SEIG and DSTATCOM parameters.

In this study, a 15 kW, 400 V three-phase induction generator is used. The SEIG parameters are obtained using experimental methods, including no-load, locked-rotor, and DC tests. The value of the star-connected excitation capacitor is selected as 270 μF to obtain a voltage of 440 V under no-load test conditions.

In this work, the DSTATCOM filter inductor is approximately computed as 5 mH by selecting a peak-to-peak current ripple ($i_{\mathrm{crpp}}$) of 6% [19]:

(A1) $$L_{f}=\frac{\sqrt{3}mV_{\mathrm{dc}}}{12af_{s}i_{\mathrm{crpp}}}=\frac{\sqrt{3}\times 1\times 700}{12\times 1.15\times 10^{4}\times (0.06\times 28.79)}≅5\;\mathrm{mH}$$

where m is the modulation index, $V_{\mathrm{dc}}$ is the DC bus voltage, a is the over-loading factor, and $f_{s}$ is the sampling frequency.

The reactive power of the star-connected three-phase capacitor bank in Figure 1 is:

(A2) $$Q_{C_{Y}}=\frac{V_{C_{Y}}^{2}}{X_{C_{Y}}}=\frac{{\left(400/\sqrt{3}\right)}^{2}}{\frac{1}{2\pi \times 50\times 270\times 10^{-6}}}=4523.9\;\mathrm{VAr}$$

The active current component of SEIG is obtained as:

(A3) $$I_{P}=\frac{P_{\mathrm{SEIG}}}{\sqrt{3}V_{L}}=\frac{15000}{\sqrt{3}\times 400}=21.65\;A$$

Its reactive current component is then obtained as:

(A4) $$I_{Q}=\sqrt{I_{rated}^{2}-I_{P}^{2}}=18.97\;A$$

The reactive power requirement to sustain the terminal voltage of the SEIG under full-load conditions is expressed as:

(A5) $$Q_{\mathrm{SEIG}}=\sqrt{3}V_{L}I_{Q}=13142.8\;\mathrm{VAr}$$

A 4.523 kVAr star-connected capacitor bank is connected at the SEIG terminals to ensure self-excitation as given in Equation (A2). Additionally, the reactive power of the load is assumed to be 7 kVAr. Therefore, the total reactive power of DSTATCOM is:

(A6) $$Q_{\mathrm{statcom}}=Q_{\mathrm{load}}+Q_{\mathrm{SEIG}}-Q_{C_{Y}}=15619.8\;\mathrm{VAr}$$

The compensating reactive current can be computed as:

(A7) $$I_{\mathrm{comp}}=\frac{Q_{\mathrm{statcom}}}{\sqrt{3}V_{L}}=22.54\;A$$

In general, it is sufficient that the power supplied by the DSTATCOM (ε) is within 10% of the total power of the system under transient conditions. In this work, the maximum transient DC bus voltage drop is constrained to 5%. Based on this principle, the energy requirement of the DC bus capacitor during transient recovery is:

(A8) $$\Delta E_{\mathrm{dc}}=\varepsilon \sqrt{3}V_{L}(aI_{\mathrm{comp}})t=\frac{1}{2}C_{\mathrm{dc}}(V_{\mathrm{dc}1}^{2}-V_{\mathrm{dc}2}^{2})$$

where

ε: ratio of the power provided by DSTATCOM to the total power

$V_{L}$: line voltage

a: overloading factor

t: duration of voltage dip (typically 30 ms)

$C_{\mathrm{dc}}$: DC bus capacitor value

$V_{\mathrm{dc}1}$, $V_{\mathrm{dc}2}$: DC bus voltages before and after transient.

Assuming ε=0.1, $V_{L}=400\;V$, a=1.15, $I_{\mathrm{comp}}=22.54\;A$, t=30 ms, $V_{\mathrm{dc}1}=700\;V$, and $V_{\mathrm{dc}2}=665\;V$, the value of $C_{\mathrm{dc}}$ is calculated as 2255.4 μF using Equation (A8). Its value can be selected as 2500 μF in experiments.
